# Clinical Oncology, 2nd edn

**DOI:** 10.1054/bjoc.2000.1517

**Published:** 2000-12

**Authors:** R Souhami

**Affiliations:** Royal Free and University College Medical School, University College London, Gower Street, London, WCIE 6BT, UK


schemata of molecular mechanisms of disease, would be welcome – perhaps in the next edition.

One of us had reviewed the first edition of *Diseases of the Breast* in 1996 and was very impressed – the second edition is as impressive and the content is thoroughly updated. It can therefore be repeated that 'this book deserves a place on the desk of anyone interested in the subject'.

*Jayant S Vaidya, Mohammed Keshtgar and Michael Baum*
Department of Surgery, University College London,
67–73 Riding House Street, London W1P 7LD, UK

doi: 10.1054/ bjoc.2000.1517, available online at http://www.idealibrary.com on IDEAL®

### Clinical Oncology, 2nd edn

MD Abeloff, JO Armitage, AS Lichtes and JE Niederhuber

Churchill Livingstone: Philadelphia

This is the second edition of this large textbook, which first appeared in 1995. It is one of several books that aim to give a comprehensive account of the science, clinical features and management of cancer.

The growth of electronic libraries and reference systems in the 1990s worried medical publishers, particularly with respect to books like this. Would clinicians continue to use them? It turns out that they do. The reasons are obvious. It's much easier to look something up quickly in a book than to turn on your computer and struggle through complex sorting procedures to find information that you then have to read on a screen. Books are easy to browse, to dip into, to find something quickly, to read on a train (although at 4.5 kg you wouldn't want this one to fall out of an overhead luggage rack). A good book, writen to *educate* rather than to review current knowledge, has a different purpose from a collection of reviews. This one is aimed at trainee and practising oncologists, as well as non-specialists. It is a work of instruction and reference with a wide audience. For the editors and publishers this means achieving consistency of style and content. They have succeeded admirably.

To start with the basics. It is 3000 pages long has 223 contributors, nearly all of whom are from North America. It is nicely printed on paper with little see-through and with figures, tables and colour prints that are, in the main, well thought out and with a certain consistency and logic from chapter to chapter. The general layout is conventional. It starts with the scientific underpinnings of cancer medicine, moves on to the general principles of management. It correctly places supportive care early in the book rather than after the sections on specific cancers that follow.

I always find the science parts of these books less useful than the clinical. They are usually mini-reviews and often do not deal conceptually with the subject at a level which will really engage the reader. The authors, basic scientists of high repute, often show little understanding of what the reader will wish to know, or where he or she might have difficulty in understanding. If the function of these sections is to teach and to explain, the text must concern itself with the clear exposition of principles. In this book many of the contributions are first-rate from this point of view. These chapters have good diagrams to explain difficult ideas. It's curious, though, that there is cumulatively much more space devoted to the science of potential biological treatments – most of which are of uncertain value – than there is to the scientific basis of drug treatment – which often works. As usual there are hundreds of references in these and the other chapters. These have become an obsession in medical writing. It seems that you can't write that patients are anxious about the diagnosis of cancer without giving three references. Who uses these lists? Why don't we limit ourselves, in textbooks of medical education and practice, to references to really good reviews or essential original articles?

The sections on the general aspects of cancer management – metastases, effusions, cord compression, paraneoplastic syndromes, nausea, cachexia, fever – are excellent. Here, as elsewhere in the book, there are summary boxes and algorithms which will help the physician, especially the trainee. The advice on diagnosis and management is clear and written concisely. I found myself occasionally at odds with some of the recommendations. Mostly this was when advice was being given about treatment, in situations where there is no chance of cure, without the option of not treating being considered. This remark will doubtless be seen as a typical example of UK nihilism and attributed to our health care system. It isn't. It's good practice not to give active treatment sometimes.

How does a reviewer give a fair account of the remaining 2000 pages of the specific tumours? I had this book for 6 months before writing this review – to the immense frustration of the Editor of the *Journal*. The reason was that I used it to look things up when I wasn't sure of my facts. It's a good test of whether a book actually works I think. It seldom let me down. I found it easy to find my way around. I read the sections on lung cancer, lymphoma, bone and soft-tissue sarcoma and childhood cancer thoroughly, and individual problems in many of the other sections. I found the description of the clinical features to be very good, the advice about diagnosis and staging to be sensible and straightforward, and the approach to management to be up-to-date and clear. There is a tendency for chemotherapy to be given a greater weight in some cancers than the results actually justify, but this probably reflects a feature of North American practice. There was one area of real confusion and that is in the discussion of round cell sarcomas of bone. This has found its way into two chapters, neither of which deals with the management well.

The graphic illustration is clear, the clinical algorithms are useful. The tables of chemotherapy regimens are highly selected (they always are) and reflect the authors' own preoccupations, but those that I looked at in detail seemed to me to be a fair summary of the most useful modern regimens.

In summary, this is a first-rate book. I congratulate the Editors. I know what a task it must have been to ensure consistency of style and content and to achieve a balance between the chapters. It will be in every oncologist's library. In my view it is one of the best big books on cancer.

*Robert Souhami*
Royal Free and University College Medical School,
University College London,
Gower Street,
London WCIE 6BT, UK

*© 2000 Cancer Research Campaign*

